# *MicroRNA-34a*/IL-6R pathway as a potential therapeutic target for ovarian high-grade serous carcinoma

**DOI:** 10.18632/oncotarget.27117

**Published:** 2019-08-06

**Authors:** Ryo Yokomizo, Nozomu Yanaihara, Noriko Yamaguchi, Misato Saito, Ayako Kawabata, Kazuaki Takahashi, Masataka Takenaka, Kyosuke Yamada, Jason Solomon Shapiro, Aikou Okamoto

**Affiliations:** ^1^ Department of Obstetrics and Gynecology, The Jikei University School of Medicine, Minato-ku, Tokyo 105-8461, Japan

**Keywords:** ovarian high-grade serous carcinoma, *miR-34a*, IL-6R, STAT3, p53

## Abstract

Accumulating evidence has indicated that microRNAs play a critical role in the pathogenesis of human cancers. *microRNA-34a* (*miR-34a*) has been shown to be a key regulator of tumor suppression by targeting several cancer-related signals, including the interleukin-6 receptor (IL-6R)/Signal Transducers and Activator of Transcription 3 (STAT3) signaling pathway. Previously, we determined that *miR-34a* expression was frequently reduced in high-grade serous carcinoma (HGSC), the major subtype of epithelial ovarian cancer (EOC). Considering that the IL-6R/STAT3 signaling pathway is upregulated and believed to be a potential therapeutic target in EOC, we investigated the biological significance of reduced *miR-34a* expression in HGSC with regard to IL-6R signaling. Additionally, we evaluated the viability of *miR-34a* as a therapeutic application for HGSC both *in vitro* and *in vivo*. Accordingly, we found that the ectopic expression of *miR-34a* significantly reduced tumor proliferation and invasion through downregulation of IL-6R expression, suggesting that reduced *miR-34a* expression might play an important role in the malignant potential of HGSC through upregulation of the IL-6R/STAT3 signaling pathway. Moreover, we demonstrated that replacement of *miR-34a* reduced tumorigenicity of HGSC *in vivo*. Therefore, this study may provide the rationale for *miR-34a* replacement as a promising therapeutic strategy for HGSC.

## INTRODUCTION

Ovarian cancer is a lethal gynecological malignancy that accounts for 5%–6% of all cancer-related deaths. In 2012 alone, a total of 238,700 new cases had been identified with 151,900 cases succumbing to mortality [1]. Epithelial ovarian cancer (EOC), the most common type of ovarian cancer, has been classified into the following subtypes according to histopathological findings: high-grade serous (HGSC), low-grade serous, mucinous, endometrioid, and clear cell carcinoma. Given that EOC generally develops with few specific symptoms, majority of the patients are diagnosed at advanced stages [2]. Therefore, diagnosing EOC at an early stage is a need that remains clearly unmet [3]. The standard treatment for EOC has been primary debulking surgery aiming for no macroscopically identifiable residual tumor followed by adjuvant platinum–taxane-based combination chemotherapy. Recently, molecular targeted therapies, including bevacizumab, poly ADP-ribose polymerase (PARP) inhibitors, and anti-programmed cell death (PD)-1 antibodies, have been helping to overcome the poor prognosis of EOC [4, 5]. Given that the clinical benefits obtained through these therapies are believed to be associated with specific molecular aberrations, identification of reliable biomarkers through molecular testing is warranted for clinical application. In the SOLO2/ENGOT-Ov21 trial, which aimed to investigate the efficacy of PARP inhibitor, clinical benefits were found in patients bearing germline *BRCA1/2* mutations, indicating that mutation testing for *BRCA1/2* should be considered in part of clinical practice [4, 6]. Similarly, microsatellite instability testing has been offered to individuals with EOC considering that it has been shown to reflect the extent of tumor mutational burden and is expected to be a predictive biomarker for superior response to anti-PD-1/PD-L1 antibodies [7]. Although the development of these molecular targeted therapies has shed light on novel treatment options for EOC, their efficacy is still limited and mortality in EOC cases with advanced stages remains substantially problematic.

Currently, accumulating evidence has indicated that microRNA (miRNA) plays a critical role in the pathogenesis of human cancers, including EOC [8–10]. Thus far, *miRNA-34a* (*miR-34a*) has been shown to be a key regulator of tumor suppression by controlling factors related to cell biology, such as proliferation, invasion, apoptosis, and chemoresistance [11, 12]. Corney et al. revealed that the *miR-34* family, which was downregulated in EOC harboring *p53* mutations, could promote tumor progression by regulating MET expression in EOC [13]. Previously, we identified unique miRNA expression pro-files that could discriminate ovarian cancer histotypes with frequently reduced *miR-34a* expression in HGSC [14]. Recently, Rokavec et al. showed that interleukin-6 receptor (IL-6R), which was a direct target of *miR-34a*, was repressed via IL-6R/signal transducer and activator of transcription (STAT) 3/*miR-34a* feedback loop in colorectal cancer [15]. Considering that the IL-6R/STAT3 signaling pathway is upregulated and believed to be a potential therapeutic target in EOC [16, 17], we hypothesized that the reduced *miR-34a* expression may play an important role in the pathogenesis of HGSC through derepression of the IL-6R/STAT3 signaling pathway.

This study therefore aimed to investigate the biological significance of reduced *miR-34a* expression in HGSC and evaluate the viability of *miR-34a* as a therapeutic application for HGSC via a series of *in vitro* and *in vivo* experiments. In addition, we evaluated the relationship between *miR-34a* expression and clinicopathological characteristics, including IL-6R expression status, in patients with HGSC.

## RESULTS

### Involvement of *miR-34a* in the pathogenesis of HGSC

**Figure 1:**
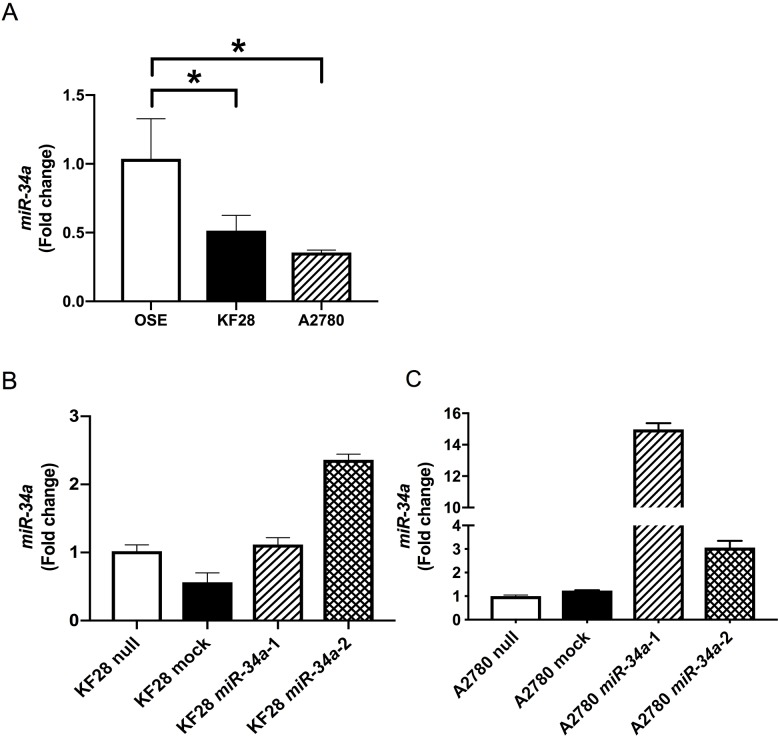
Establishment of HGSC cell lines with stable *miR-34a* overexpression *miR-34a* expression was quantified using the comparative method in real-time RT-PCR analysis. **(A)** *miR-34a* expressions were significantly lower in HGSC cell lines (KF28 and A2780) than in OSE cell line **(B, C)** HGSC cell lines with stable *miR-34a* overexpression were established in KF28 (B) and A2780 (C) cells. ^*^ means *P* < 0.05.

**Figure 2:**
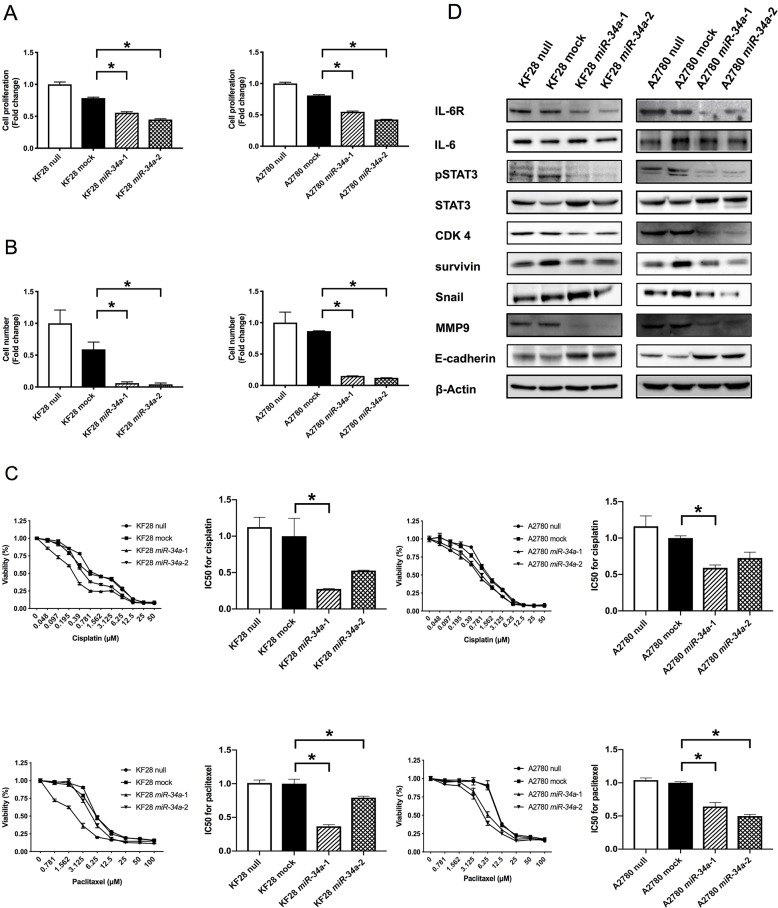
Involvement of *miR-34a* in HGSC pathogenesis *in vitro*. **(A)** Cell proliferation ability was analyzed using the MTS assay in KF28 and A2780 cells. HGSC cell lines with *miR-34a* overexpression had lower cell proliferation ability compared with negative control cells. **(B)** Cell invasion ability was analyzed using the invasion assay in KF28 and A2780 cells. HGSC cell lines with *miR-34a* overexpression had lower cell invasion ability compared with negative control cells. **(C)** Responsiveness of HGSC cell lines to cytotoxic agents was analyzed using the cytotoxicity assay in KF28 and A2780 cells for cisplatin and paclitaxel. *miR-34a-*transfected HGSC cells showed significantly greater cell death due to cytotoxic agents compared with negative control cells. **(D)** Protein expression of HGSC cell lines was analyzed using Western blot analysis. Forced expression of *miR-34a* reduced IL-6R expression affecting downstream of the IL-6R/STAT3 signaling pathway. β-Actin was used as loading control. ^*^ means *P* < 0.05.

To examine the biological significance of reduced *miR-34a* expression in HGSC, we conducted miRNA mimic-based replacement experiments using two HGSC cell lines, KF28 (*p53* dominant-negative mutant) and A2780 (*p53* wild type), in which *miR-34a* expression was significantly lower compared with noncancerous ovarian surface epithelium (OSE) (Figure 1A). We first measured *in vitro* proliferation and invasion using cells with stable *miR-34a* overexpression (Figure 1B-1C). Accordingly, HGSC cell lines with *miR-34a* overexpression had reduced cell proliferation and invasion compared with either cells expressing a nontargeting control (mock) or parental cells (null) (Figure 2A-2B, Supplementary Figure 1). We further examined the responsiveness of these cell lines to cytotoxic agents, including cisplatin and paclitaxel. Both *miR-34a-*transfected HGSC cells showed significantly greater cell death in response to either cisplatin or paclitaxel compared with mock and null controls (Figure 2C).

Next, we addressed whether *miR-34a* in HGSC could target IL-6R, which has been shown to be highly expressed in EOC [16, 18]. Accordingly, *miR-34a* overexpression reduced IL-6R expression and subsequently decreased STAT3 phosphorylation at tyrosine 705 without affecting overall levels of IL-6 or STAT3 (Figure 2D, Supplementary Figure 3). In addition, CDK4 and survivin, mediators of cell proliferation, decreased in HGSC cell lines with stable *miR-34a* overexpression (Figure 2D, Supplementary Figure 3). Furthermore, *miR-34a* overexpression increased E-cadherin expression and was associated with reduced Snail and MMP9 expression, indicating that epithelial–mesenchymal transition (EMT) was inhibited in these cell lines.

To clarify the functional interaction between *miR-34a* and IL-6R in HGSC, we investigated whether restoration of IL-6R expression could reverse the *miR-34a*-induced inhibitory effect on cell proliferation in HGSC cell lines. Accordingly, IL-6R overexpression in A2780 cells co-transfected with a *miR-34a* mimic partially rescued suppression of cell proliferation (Supplementary Figure 2A).

### Involvement of *miR-34a* in HGSC tumorigenicity *in vivo*

**Figure 3:**
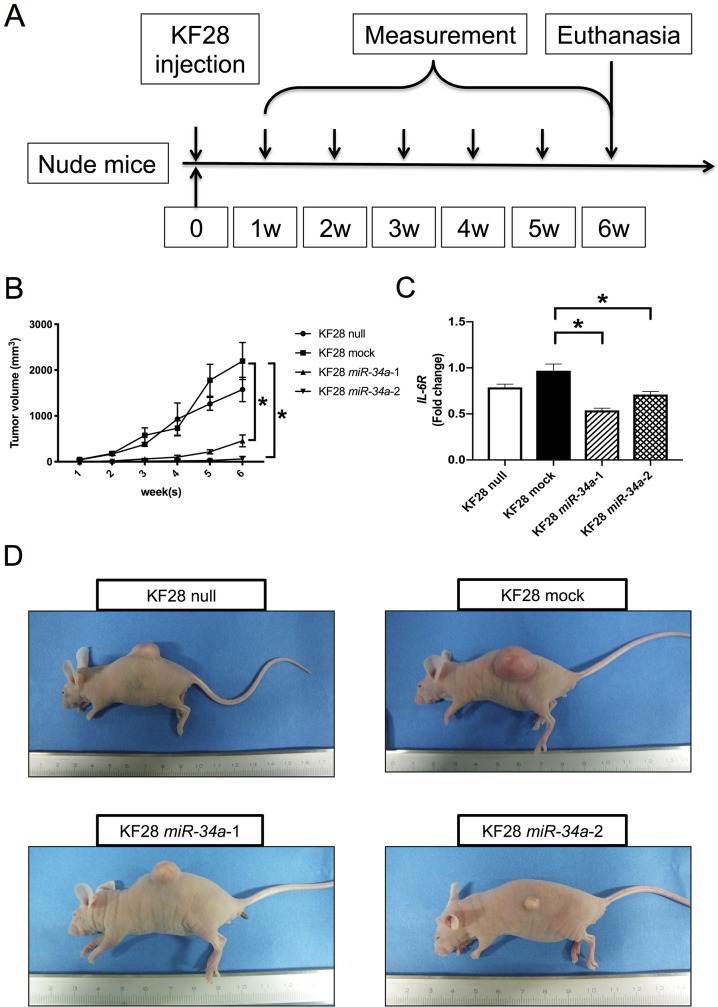
Replacement of *miR-34a* inhibiting HGSC tumorigenicity *in vivo*. **(A)** KF28 cells were injected subcutaneously into nude mice. Tumor growths were monitored up to 6 weeks. **(B)** An overall reduction in the tumor volume was observed in mice inoculated with KF28 cells having stable *miR-34a* overexpression. **(C)** *IL-6R* mRNA expression of the tumors were quantified using the comparative method in real-time RT-PCR analysis. **(D)** Representative images of the gross tumor morphology showing tumor formation in mice. ^*^ means *P* < 0.05.

To further evaluate the role of *miR-34a* in HGSC tumorigenicity, KF28 cells with stable *miR-34a* overexpression were injected subcutaneously into BALB/c nude mice, after which tumor growth was monitored for up to 6 weeks (Figure 3A). In the KF28 null, mock, and *miR-34a-1* groups, 5 mice were used and in the KF28 *miR-34a-2* group, 3 mice were used because tumor formation was not observed in 2 out of 5 mice. Mice injected with KF28 cells stably expressing *miR-34a* showed an overall reduction in tumor burden compared with those in the null or mock groups (Figure 3B). Consistent with increased *miR-34a* levels, *IL-6R* expression was significantly reduced in KF28 cells overexpressing *miR-34a* (Figure 3C). Representative images of subcutaneous tumor xenographs are shown in Figure 3D.

### *miR-34a* expression in patients with HGSC

**Figure 4:**
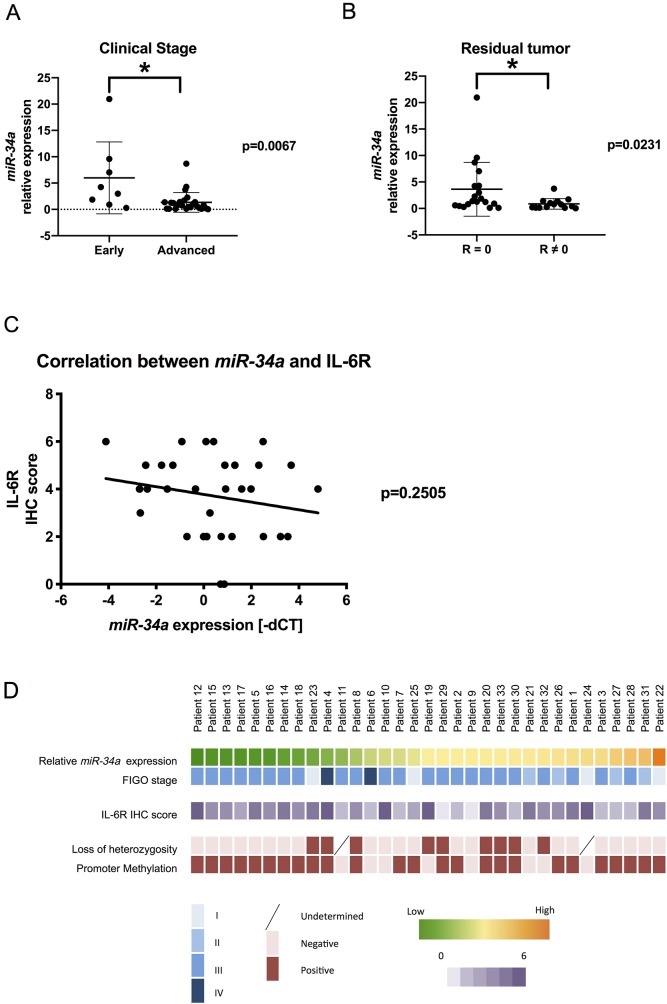
Clinical relevance of *miR-34a* in patients with HGSC *miR-34a* expression was quantified using the comparative method in real-time RT-PCR analysis. **(A)** Patients with advanced-stage disease had significantly lower *miR-34a* expression levels than those with early-stage disease. **(B)** Patients with residual tumors had significantly lower *miR-34a* expressions than those without residual tumors after surgery. R: residual tumors. **(C)** Linear regression analysis of the correlation between *miR-34a* expression (−ΔCT) and IL-6R IHC score showed an inverse trend. **(D)** Clinicopathological characteristics of patients were described using a heat map. ^*^ means *P* < 0.05

Considering our findings *in vitro* and *in vivo*, we were motivated to investigate the clinical relevance of *miR-34a* in patients with HGSC. We examined primary specimens derived from 33 patients with HGSC and assessed *miR-34a* expression using real-time reverse transcription-polymerase chain reaction (RT-PCR) analysis. Clinical characteristics of patients are presented in Table 1. Accordingly, patients with advanced-stage disease (stages III and IV) had significantly lower *miR-34a* expression than those with early-stage disease (stages I and II) (Figure 4A). Additionally, patients without residual tumor after initial surgery showed a significant reduction in *miR-34a* expression (Figure 4B). We subsequently measured IL-6R expression in the same cohort using immunohistochemical analysis. Although no statistical correlation was found, there was a trend of inverse correlation between *miR-34a* expression and IL-6R immunohistochemistry (IHC) score (Figure 4C). Next, we explored the underlying mechanisms for reduced *miR-34a* expression in the clinical specimens. Both the deletion of the *miR-34a* genomic locus (1p36.22) and hypermethylation of the promoter region have been reported as possible mechanisms for *miR-34a* downregulation in EOC [19]. Using a TaqMan PCR-based method for copy number analysis, loss of heterozygosity for the *miR-34a* genomic region was observed in 9 patients (29.0 %) within our cohort (Figure 4D, Supplementary Table 1). To examine the CpG methylation status of the *miR-34a* promoter lesion, the MethyLight analysis was performed. Accordingly, *miR-34a* promoter methylation was observed in 25 patients (75.8 %) with percentage of methylated reference values ranging from 0.58 to 44.26 (Figure 4D, Supplementary Table 1).

**Table 1:** Clinicopathological characteristics of the 33 patients with HGSC

Parameters	n = 33
Patient age (years, mean ± SD)	55.2 ± 7.9
BMI (kg/m^2^, mean ± SD)	19.3 ± 2.9
FIGO stage	
I (%)	4 (12.1)
II (%)	4 (12.1)
III (%)	23 (69.7)
IV (%)	2 (6.1)
Follow-up period [months, median (IQR)]	27 (11-72)
Recurrence or progression (%)	9 (27.3)
Platinum sensitive (%)	7 (77.8)
Platinum resistant (%)	2 (22.2)
Death at the observation time point (%)	5 (15.2)
Residual tumor after initial surgery	
R = 0 (%)	19 (57.6)
R ≠ 0 (%)	14 (42.4)

SD, standard deviation; BMI, body mass index; FIGO, International Federation of Gynecology and Obstetrics; IQR, interquartile range; R, residual tumors.

## DISCUSSION

Accumulating evidence from miRNA profiling has demonstrated that miRNAs play essential roles in ovarian carcinogenesis and impact different clinical aspects [20, 21]. Upregulated miRNAs that are present in cancer cells and contribute to cancer development by inhibiting tumor suppressor genes are considered oncogenic miRNAs, whereas downregulated miRNAs that suppress cancer development by inhibiting proto-oncogenes are known as tumor suppressor miRNAs. In the HGSC cell lines used herein, ectopic expression of *miR-34a* significantly reduced proliferation and invasion abilities through the downregulation of IL-6R expression and downstream STAT3 signaling, suggesting that redu-ced *miR-34a* expression might play an important role in the malignant potential of HGSC cells. This phenotype was observed regardless of the original *p53* status, thus establishing *miR-34a* as an independent tumor suppressor miRNA. Since STAT3 activation is induced by phosphorylation at a critical tyrosine residue (tyrosine 705), and this phosphorylation is mainly regulated in the STAT3 signaling pathway [22] we have investigated STAT3 phosphorylation at tyrosine 705 in the present study. In addition, as cell confluence might affect STAT3 phosphorylation [23] we uniformed the cell confluence at approximately 80% in null, mock, and *miR-34a* overexpression cells for Western blot analysis. Based on this setting, downregulation of pSTAT3 expression may not be affected by cell confluence but triggered by *miR-34a* replacement. It is also known that STAT3 activates its expression with binding to own promoter [24], and therefore downregulation of the STAT3 signaling pathway may lead to downregulation of total STAT3 expression. However, in the present study, total STAT3 expressions were not substantially changed in *miR-34a* overexpression cells. We previously reported that downregulation of the IL-6R/STAT3 signaling pathway did not affect total STAT3 expression in three ovarian cancer cell lines, as consistent with this study [17, 25]. These findings suggest that positive feedback regulation of STAT3 may be tissue and/or condition dependent. An earlier finding showing that the IL-6R/STAT3/ *miR-34a* feedback loop promoted EMT in colorectal cancer prompted us to investigate the existence of the same feedback loop in HGSC [15]. However, downregulation of IL-6R by siRNA did not induce *miR-34a* expression in the HGSC cell line, A2780 (Supplementary Figure 2B), suggesting that IL-6R/STAT3/*miR-34a* feedback is tumor specific. Corney et al. demonstrated that frequent *miR-34* family downregulation was correlated with EOC metastasis [13]. In addition, a recent report showed a significant association between decreased *miR-34a* expression and worse prognosis in patients with EOC [26, 27]. Consistent with the aforementioned reports, we also identified that reduced *miR-34a* expression in HGSC was associated with advanced clinical stage. Taken together, these results indicated that *miR-34a* might be involved in HGSC pathogenesis through its effects on tumor progression [21]. The underlying mechanisms for reduced *miR-34a* expression in HGSC can be largely explained by *p53* loss-of-function mutations, which were detected in 96% of the patients with this EOC subtype [13, 27, 28]. However, consistent with previous reports [13, 27], we also observed a wide range of promoter methylation and/or copy number alterations at the *miR-34a* locus in our cohort, although no clear association between either methylation or copy number change and *miR-34a* expression was observed.

Bioinformatic approaches and *in vitro* experiments have validated various molecules, including MET, Snail, E2F3a, and histone deacetylase 1 (HDAC1), as direct targets of *miR-34a* in EOC [13, 26, 29, 30]. Reduced *miR-34a* expression may affect the invasive behavior of EOC by inducing EMT via alleviating the repression of MET or Snail [13, 26]. Similarly, *miR-34a* has been shown to suppress ovarian cancer cell proliferation and chemoresistance by controlling HDAC1 expression [30]. Here we demonstrate that *miR-34a* exerted tumor-suppressive effects in HGSC by regulating the IL-6R/STAT3 signaling pathway and the associated expression of Snail, MMP9, CDK4, and survivin. These IL-6R/STAT3-mediated regulatory proteins could perhaps also be direct targets of *miR-34a* as shown in earlier studies [11, 31]. The ability of *miR-34a* to directly bind to the 3′-UTR of IL-6R has been demonstrated in the context of colorectal cancer [15]. The present study provides independent evidence that *miR-34a* regulates the IL-6R/STAT3 pathway in ovarian cancer by demonstrating that IL-6R reexpression is able to mitigate the effects of *miR-34a* mimetic oligos (Supplementary Figure 2A). Although the involvement of the IL-6R/STAT3 signaling pathway in EOC has been explored with regard to tumor progression and chemoresistance [16, 17, 25, 32–34], the use of antibodies (siltuximab and tocilizumab) targeting this signaling pathway has not generated clear clinical relevance [32, 33, 35]. Alternative approaches for repressing IL-6R signaling in the context of HGSC are thus needed.

MiRNA-based therapy has great potential for becoming a more powerful tool in cancer treatment by simultaneous modulation of multiple genes involved in cancer-related signaling pathways. Based on our findings from *in vitro* analyses, we further investigated the viability of *miR-34a* as a therapeutic application for HGSC *in vivo*. The results obtained from mice transplanted with stable *miR-34a* overexpression xenographs showed that replacement of *miR-34a* could effectively reduce tumorigenicity. Furthermore, *IL-6R* mRNA expression, which is directly regulated by *miR-34a,* was significantly reduced in KF28 cells overexpressing *miR-34a* (Figure 3C). We also demonstrated that pSTAT3 expression level in KF28 cells overexpressing *miR-34a* was downregulated compared with that in control by Western blot analysis (Supplementary Figure 4). However, the effect of *miR-34a* replacement on STAT3 activation was not significant compared with that on *IL-6R* mRNA expression. We hypothesized the etiologies accounting for this difference owing to the following reasons. First, there might be survival advantages in this *in vivo* experiment. Namely, the STAT3 signaling pathway is essential to survive so that its activation in tumors obtained from survived mice may be maintained to some extent in *miR-34a* replacement group. This explanation can be supported by the fact that tumors were harvested after a long duration since the transplantation of the cancer cells. Second, the STAT3 signaling pathway is regulated by many factors other than IL-6R; therefore, *miR-34a* cannot strongly affect STAT3 activation compared with *IL-6R* mRNA expression that is directly regulated by *miR-34a*. These etiologies may produce the difference in the effect of *miR-34a* on its correlating targets. A previous study has shown that the soluble form of IL-6R not only potentiates the effects of secreted IL-6 but also widens the range of cells affected by IL-6 [36]; in this regard, *miR-34a* may affect the soluble form of IL-6R and can be a potential rationale for reducing the tumorigenicity.

In our animal model, *miR-34a* was stably integrated into the genome of the tumor cells. The main obstacle has been the low efficiency of exogenous miRNA delivery into cancer cells using currently available methods [11]. Further research is required to develop more efficient strategies to reexpress *miR-34a*. In other types of tumors, *miR-34a* replacement therapeutic strategies have also been investigated in preclinical setting. For multiple myeloma, the combination of *miR-34a* with other anticancer agents strongly inhibits tumor growth and appears as a promising strategy [37]. Combined with radiotherapy, *miR-34a* can improve the efficacy of lung cancer radiotherapy by inducing senescence via targeting c-Myc [38]. Furthermore, the development of bioengineered RNAs as novel therapeutic agents has been paid attention, and bioengineered *miR-34a* prodrug has been demonstrated to be effective in controlling osteosarcoma tumor growth [39]. To date, only one phase I clinical trial has demonstrated the oncosuppressive effects of *miR-34a* in patients with solid tumors [40]. However, adverse effects, such as myelosuppression and liver damage, were also observed. Although unmet requirements still have to be resolved before *miR-34a* can be used in clinical practice, improvements in drug delivery systems and management of adverse effects may make it applicable for clinical use in the near future.

In conclusion, we show that reduced *miR-34a* expression is strongly correlated with HGSC pathogenesis through its regulation of the pro-inflammatory IL-6R/STAT3 pathway and its subsequent targets. Accordingly, the present study provides the rationale for *miR-34a* replacement as a promising therapeutic target strategy for HGSC. Nonetheless, further work is required to establish a safe and efficient delivery system for *miR-34a* and similar tumor-suppressive miRNAs.

## MATERIALS AND METHODS

### Clinical samples and cell lines

This study involved surgically obtaining tumor specimens of primary ovarian cancer from patients receiving treatment at Department of Obstetrics and Gynecology, The Jikei University School of Medicine. The Ethics Review Committee of The Jikei University School of Medicine approved of the study protocol [approval number: 27-076(7961), 28-063(8326)], and all patients provided written informed consent prior study participation. Tumors were staged according to the International Federation of Gynecology and Obstetrics staging system (2014). This study used two ovarian serous carcinoma cell lines, KF28 and A2780. KF28, a single-cell clone of the human ovarian serous carcinoma cell line [41], was obtained as a kind gift from Dr. Kikuchi Y (Ohki Memorial Kikuchi Cancer Clinic for Women, Saitama, Japan), whereas A2780 cells were purchased from KAC Inc. (Tokyo, Japan). KF28 was negative for mycoplasma contamination tested using Cycleave™ PCR Mycoplasma Detection Kit (Takara Bio Inc., Shiga, Japan). KF28 and A2780 cells were cultured in RPMI-1640 (Sigma-Aldrich, Tokyo, Japan) containing 10% fetal bovine serum (FBS). As a normal control, we used human OSE cell lines established as described previously [42]. OSE cells were maintained in RPMI-1640 (Sigma-Aldrich, Tokyo, Japan) containing 10% FBS.

### Establishment of HGSC cell lines with stable *miR-34a* overexpression

Stable *miR-34a* overexpression clones were established in KF28 and A2780 cells through plasmid vector transfection and Lipofectamine™ 2000 Transfection Reagent (Invitrogen). As a selectable marker, neomycin resistance gene was used and cultured with G-418-containing medium, after which the surviving colonies expressing the neomycin resistance gene were collected.

### RNA and DNA extraction

Freshly excised surgical specimens were stored in RNAlater™ Solution (Thermo Fisher Scientific, MA, USA) at 4°C for 24 h and subsequently frozen at −80°C prior to RNA extraction. We used either the manual method using TRIzol reagent (Invitrogen, Carlsbad, CA) according to the manufacturer’s instruction or the automated method using the gentleMACS™ Octo Dissociator with Heaters (Miltenyi Biotec, Gladbach, Germany) for homogenization and the Maxwell™ RSC simplyRNA Tissue Kit (Promega Corporation, Madison, WI, USA) for total RNA extraction. For DNA extraction, freshly excised surgical specimens were stored at −80°C. Two extraction methods were utilized, including the manual method using the Gentra Puregene Tissue Kit (Qiagen, Venlo, Netherlands) according to the manufacturer’s instruction and the automated method using the gentleMACS™ Octo Dissociator with Heaters (Miltenyi Biotec, Gladbach, Germany) for homogenization and the Maxwell™ RSC blood DNA Kit (Promega Corporation, Madison, WI, USA).

### Quantitative real-time reverse transcription-polymerase chain reaction (RT-PCR) analysis

Extracted RNAs for *IL-6R* mRNA assessment were subjected to reverse transcription using qScript cDNA SuperMix™ (Quantabio, Beverly, MA). Complementary DNAs (cDNAs) were subjected to quantitative real-time RT-PCR using PerfeCTa SYBR™ Green FastMix (Quantabio, Beverly, MA). Meanwhile, miRNAs were assessed by subjecting them to reverse transcription using the TaqMan™ MicroRNA Reverse Transcription Kit (Applied Biosystems, Foster City, CA, USA). cDNAs were subjected to quantitative real-time RT-PCR analysis using TaqMan™ Fast Advanced Master Mix (Applied Biosystems, Foster City, CA, USA). All PCR reactions were performed in 96-well plates using the StepOnePlus™ real-time PCR System (Applied Biosystems). Glyceraldehyde 3-phosphate dehydrogenase was used as an endogenous control during mRNA PCR, whereas *SNORD38B* labeled with FAM reporter dye (Applied Biosystems) was used as an endogenous control during miRNA PCR. In the experiments, parental cells or negative control were set as the reference. *miR-34a* expression was quantified using the comparative method (2^−ΔΔCT^), where CT = threshold cycle, ΔΔ CT = (CT_*miR-34a*_ − CT _*SNORD38B*_) − (CT _reference_ − CT _*SNORD38B*_).

### Transfection assay

For miRNA transfection, A2780 cells were seeded into 6-cm dishes at a density that would yield 70% confluency after 24 h and were subsequently transfected. This cell line was transfected using the mirVana™ miRNA mimic (Thermo Fisher Scientific) specific for *miR-34a* or mirVana™ miRNA mimic Negative Control (Thermo Fisher Scientific) at a final concentration of 50 nM. For siRNA transfection, Stealth RNAi™ siRNAs for *IL-6R* (Invitrogen) and negative control (Invitrogen) were used at a final concentration of 5 nM. For transfection of the *IL-6R* gene, plasmid vector pEZ-M61-IL-6R (EX-A0457-M61, GeneCopoeia™) or empty pReciever-M61 (EX-NEG-M61, GeneCopoeia™) were used at a final concentration of 1.5 nM. RNA and DNA transfection were performed using Lipofectamine™ RNAiMAX Transfection Reagent (Invitrogen) and Lipofectamine™ 2000 Transfection Reagent (Invitrogen), respectively, according to the manufacturer’s instruction.

### Western blot analysis

Cells at approximately 80% confluence were collected and subjected to Western blot analysis. Total cell lysates were prepared in 1× radioimmunoprecipitation assay lysis buffer, after which protein concentration was analyzed using the DC™ Protein Assay (Bio-Rad Laboratories, Hercules, CA, USA). Total protein was resolved on gradient NuPage 4%–12% Bis-Tris gels (Thermo Fisher Scientific). Proteins were then transferred onto membranes using an iBlot1 Gel Transfer Device (Thermo Fisher Scientific). The membranes were incubated with primary antibodies at 4°C with gentle agitation. All antibodies were diluted in Tris-buffered saline containing 0.1% Tween 20 and 5% bovine serum albumin. Horseradish peroxidase-conjugated secondary anti-rabbit or anti-mouse antibody (Cell Signaling Technology; 1:10000) was diluted in Tris-buffered saline with 0.1% Tween 20 and 5% nonfat milk for 1 h at room temperature with gentle agitation. Positive immunoreactions were detected using the ImmunoStar LD chemiluminescence system (Wako, Tokyo, Japan). Antibodies against CDK4 (clone D9G3E; 1:1000), survivin (clone 91G4B7; 1:1000), Snail (clone L70G2; 1:1000), STAT3 (clone 79D7; 1:2000), phosphorylated STAT3 (Tyr705) (cloneD3A7; 1:500), and β-Actin (clone 13E5; 1:1000) were obtained from Cell Signaling Technology (Beverly, MA), whereas those against IL-6Ra (clone C-20; 1:1000) were obtained from Santa Cruz Biotechnology (Santa Cruz, CA). Mouse monoclonal antibodies against MMP-9 (clone 56-2A4; 1:500), MDM4 (clone GR238615-19, 1:1000), and E-cadherin (clone EP700Y; 1:50000) were purchased from Abcam (Cambridge, UK). Antibodies against IL-6 (clone 21865-1-Ap; 1:1000) were obtained from Proteintech (Rosemont, IL)

### MTS assay

MTS assay was performed using the CellTiter 96 AQueous One Solution Cell Proliferation Assay kit (Promega, Madison, WI) following the manufacturer’s protocol. Briefly, MTS solution was added to each of the 96-well plates and incubated for 1 h, after which absorbance was measured at 490 nm using a microplate reader.

### Cell proliferation assay

KF28 and A2780 cells were seeded into 96-well plates at a density of 8 × 10^3^ cells per well in triplicate at the least. MTS solution was added 1 h before each of the desired time points and cells were incubated at 37°C. Data were collected as the average absorbance of the wells, and each experiment was repeated three times with values being presented as mean ± standard error of the mean (SEM).

### Invasion assay

KF28 and A2780 cells were seeded onto the top chamber of 24-well matrigel-coated polyethylene terephthalate membrane inserts with 8-mm pores (Corning, Tewksbury, MA). The bottom chamber was filled with 0.75 ml of medium with 10% FBS as a chemoattractant. Insert cups with 0.5 ml of medium but without FBS were then prepared. After incubation for 48 h, the filter membrane was fixed with 100% methanol and stained using Wright–Giemsa staining. The degree of invasiveness was quantified by counting the number of cells in at least three random fields of view per filter using ×100 magnification. All experiments were repeated three times with values being presented as mean ± SEM.

### Cytotoxicity assay

KF28 and A2780 cells were seeded into 96-well plates at a density of 1 × 10^4^ and 1 × 10^3^ cells per well, respectively, with varying concentrations of paclitaxel and cisplatin (see Figure 2). *In vitro* chemosensitivity was measured after 96 h using the MTS assay as described. Data were collected as the average absorbance of three wells in each of the three independent experiments with values being presented as mean ± SEM. The IC50 that inhibited the absorbance was defined as the paclitaxel and cisplatin concentration at 490nm to 50%.

### Immunohistochemical analysis

Immunohistochemical analysis of IL-6R expression was performed on 3-mm paraffin sections of formalin-fixed, paraffin-embedded tissues using the Ventana Discovery XT automated stainer (Ventana 20 Medical Systems, Tucson, AZ). After deparaffinization, antigen retrieval was carried out using CC1buffer (Cell Conditioning 1; citrate buffer pH 6.0, Ventana Medical Systems). Two investigators (R. Y. and J. S.S.) scored the expression levels based on the stain intensity and extent. IHC score was conducted entirely independent of all clinical variables. Similar to our previous study [30], positively stained tumor cells were graded using a semiquantitative five-category system: 0, <5% positive cells; 1, 6%–25%; 2, 26%–50%; 3, 51%–75%; and 4, 76%–100%. The intensity of positively stained tumor cells was determined using scores of 0 to 2 as follows: 0 (none), 1 (weak; intensity was less than that in the positive control), and 2 (strong; intensity was equal to or greater than that in the positive control). The addition of these two systems provided the overall score.

### Copy number assay

Chromosome 1p36.22, in which the *miR-34a* gene is located, was targeted for this analysis. RNaseP was used as the endogenous control. For TaqMan™ Copy Number Assay, 1p36.22 labeled with FAM reporter dye (Applied Biosystems) and RNaseP labeled with TAMRA (Applied Biosystems) were used. PCR was performed as described previously, after which data were analyzed using CopyCaller™ Software v2.1 (Thermo Fisher Scientific).

### MethyLight analysis

Bisulfite modification was performed using the EZ DNA Methylation-Gold Kit (Zymo Research, Orange, CA, USA) according to the manufacturer’s instructions. Two sets of primers designed specifically for bisulfite-converted DNA were used: a methylated set for the *miR-34a* promoter and a reference set, *COL2A1*, to normalize for input DNA. The specificity of the reactions for methylated DNA was confirmed separately using SssI (New England Biolabs, Frankfurt, Germany)-treated human white blood cell DNA (heavily methylated). Methyl-specific primers failed to amplify genomic DNA not subjected to bisulfite treatment. The percentage of fully methylated molecules at a specific locus was calculated by dividing the *miR-34a* promoter/*COL2A1* ratio of a sample by that of SssI-treated controls and multiplying by 100. The threshold for detectable methylation was set using a CT value < 35, whereas a CT value ≥ 35 was defined as methylation negative. Identification of CpG islands and primer sequences were obtained from Reimer et al.’s report [29], and all primer sequences are presented in Supplementary Table 2.

### Animal experiment

BALB/cSlc-nu/nu female mice aged 5 weeks were obtained from Sankyo Laboratory Co. Ltd. (Tokyo, Japan) and housed under a 12-h darkness/light cycle in an animal facility at The Jikei University School of Medicine with a controlled temperature (20°C–25°C) and humidity (40%–70%). Food and water were provided *ad libitum* throughout the study. Mice were allowed to acclimatize for 1 week, after experiments were performed. KF28 cells were injected subcutaneously into 20 mice to construct human cancer models and assess the potential availability of *miR-34a.* These 20 mice were divided into four groups and subcutaneously injected with the following cells (5 × 10^6^/in 50 μl PBS): (1) null KF28 cells (n = 5), (2) mock KF28 cells (n = 5), (3) KF28 *miR-34a*-1 cells (n = 5), and (4) KF28 *miR-34a*-2 cells (n = 5). Seven days after the subcutaneous injection of KF28 cells, tumor formations were confirmed in 18 mice (no tumor formation in two mice injected with KF28 *miR-34a*-2). Observation was maintained for 42 days, and tumor sizes were measured by diameter in three directions (diameters 1, 2, and 3) weekly. Tumor volumes were calculated using the following equation: Tumor Volume (mm^3^) = Diameter 1 (mm) × Diameter 2 (mm) × Diameter 3 (mm). On the last day, all mice were euthanized by cervical dislocation while under general anesthesia (induced using an isoflurane inhalation). The tumors were immediately excised with some portions thereof being stored at −80°C for biochemical analysis and the others being fixed in 4% paraformaldehyde for histological study. The protocol for these animal experiments was reviewed and approved by the Institutional Animal Care and Use Committee of The Jikei University (No. 2015-114) and conformed to the Guidelines for the Proper Conduct of Animal Experiments of the Science Council of Japan (2006).

### Statistical analysis

Statistical analyses were performed using Prism 8.01 software (GraphPad, Inc.). The Mann–Whitney *U* test was used to compare the means between two independent groups of *in vitro* assays, *in vivo* assays, and clinical samples. For the assessment of tumor growth in the animal model, we calculated the area under the curve corresponding to the tumor growth curve and analyzed using ordinary one-way ANOVA for multiple comparisons. Associations between clinicopathological parameters and miRNA expressions were analyzed using the chi-square test, Mann–Whitney *U* test, and linear regression analysis. Statistical significance was set at *P* < 0.05.

## SUPPLEMENTARY MATERIALS FIGURES AND TABLES



## Abbreviations

miRNA: microRNA
HGSC: high-grade serous carcinoma
PARP: poly ADP-ribose polymerase
IL-6R: interleukin-6 receptor
STAT3: Signal Transducers and Activator of Transcription 3
EOC: epithelial ovarian cancer
EMT: epithelial–mesenchymal transition
RT-PCR: reverse transcription-polymerase chain reaction
FBS: fetal bovine serum
cDNAs: complementary DNAs
SEM: standard error of the mean
CDK4: cyclin-dependent kinase 4
MMP, 9: matrix metalloproteinase, 9
IC50: concentration producing 50% inhibition
OSE: ovarian surface epithelium
HDAC1: histone deacetylase 1
FIGO: International Federation of Gynecology and Obstetrics
IHC: immunohistochemistry.
