# Supplementary material for: MicroRNA-34a/IL-6R pathway as a potential therapeutic target for ovarian high-grade serous carcinoma
Source: Oncotarget. 2019 Aug 6;10(47):4880–93. doi: 10.18632/oncotarget.27117 (PMC6690672; doi:10.18632/oncotarget.27117)
Supplement: Supplementary file 1 [file oncotarget-10-4880-s001.pdf]

MicroRNA-34a/IL-6R pathway as a potential therapeutic target for ovarian high-grade serous carcinoma

SUPPMENTARY MATERIALS

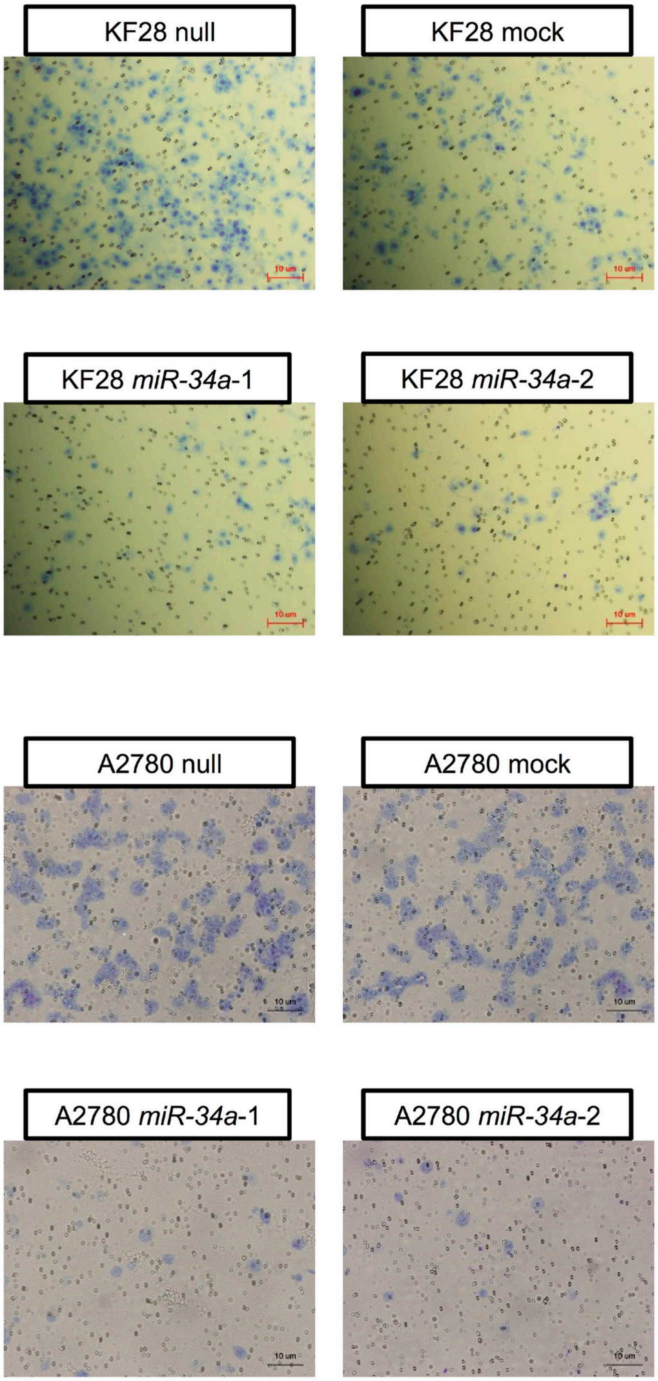

Supplementary Figure 1: Representative images of the invasion assay. Representative images of the invasion assay showing that HGSC cell lines with *miR-34a* overexpression had reduced cell invasion ability compared with negative control cells.

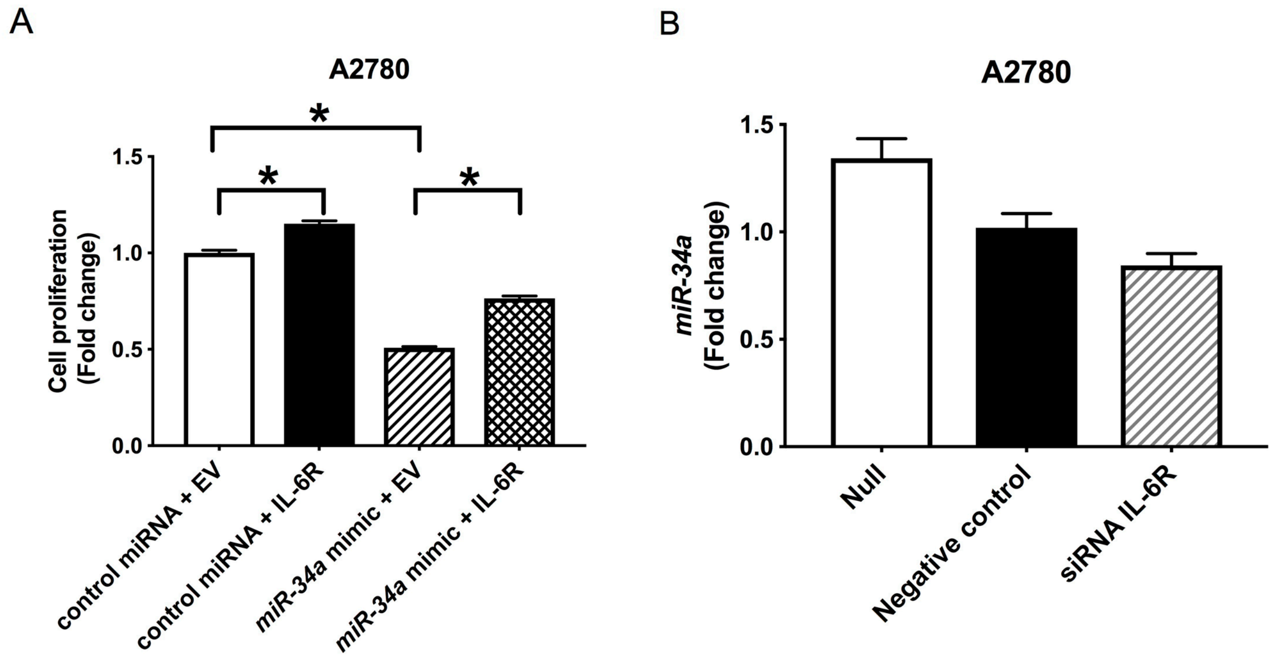

Supplementary Figure 2: Correlation between IL-6R and *miR-34a* in A2780. (A) Restored IL-6R expression partially recovered the cell proliferation ability suppressed by *miR-34a* mimic. **(B)** *miR-34a* expression was quantified using the comparative method in real-time RT-PCR analysis. IL-6R downregulation with siRNA transfection did not induce *miR-34a* expression.

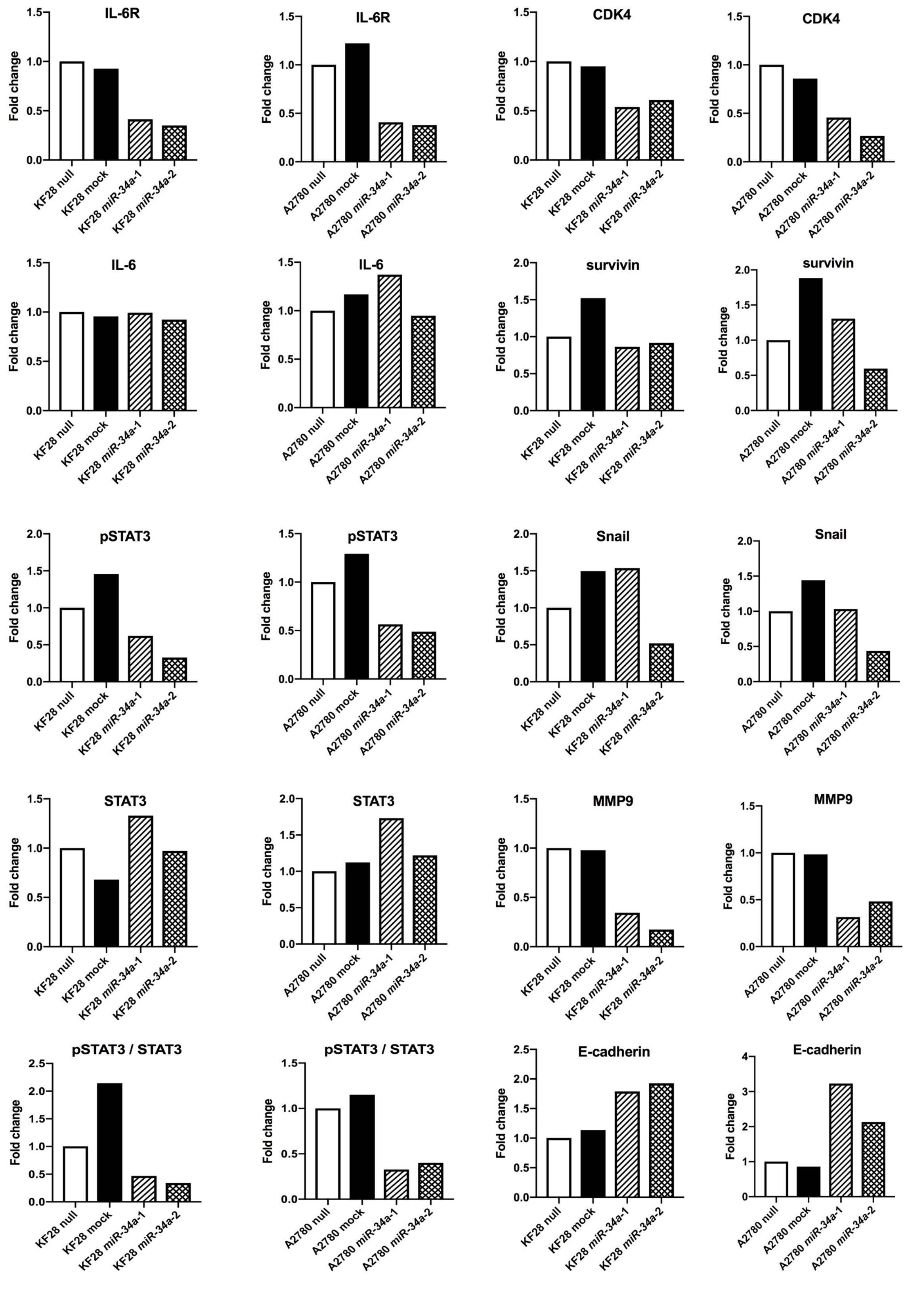

Supplementary Figure 3: Densitometric analysis of Western blot analysis. Protein bands demonstrated in Figure 2D were quantified with densitometric analysis. Each band normalized to corresponding β-Actin was indicated. The ratio of pSTAT3 to STAT3 was also indicated.

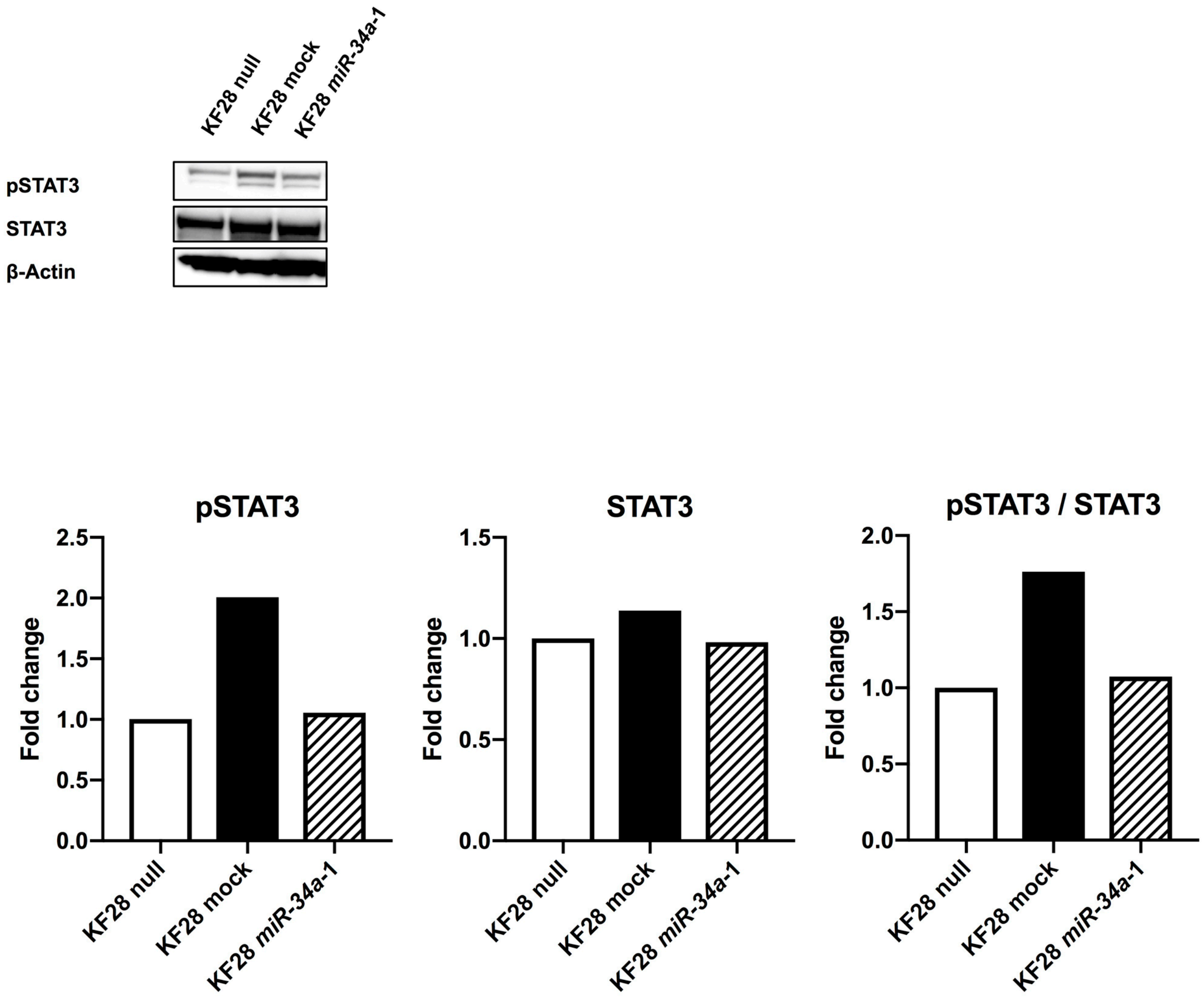

Supplementary Figure 4: Expressions of pSTAT3 and STAT3 in the tumors obtained from *in vivo* experiments. Expression of pSTAT3 and STAT3 in the tumors were analyzed using Western blot analysis. β-actin was used as loading control. Protein bands were quantified with densitometric analysis. Each band normalized to corresponding β-Actin was indicated. The ratio of pSTAT3 to STAT3 was also indicated.

Supplementary Table 1: Patient characteristics associated with *miR-34a*, IL-6R, copy number of 1p36.22, and methylation of the *miR-34a* promoter region

Patient	Age (years)	FIGO stage	Residual tumorR ≠ 0: +R = 0: −	Relative*miR-34a* expression	IL-6RIHC score	Copy number of 1p36.22loss: +normal or amplification: −	PMR (%) negative: −
1	51	III	+	3.73	5	−	5.75
2	60	III	−	1.25	2	−	19.62
3	61	III	−	4.28	2	−	2.43
4	49	IV	+	0.40	6	+	0.58
5	65	III	+	0.14	5	−	30.88
6	60	IV	+	0.75	2	−	−
7	69	III	−	0.82	2	−	4.31
8	55	III	−	0.59	4	+	8.21
9	40	III	+	1.36	0	−	−
10	63	III	+	0.80	6	−	−
11	52	III	−	0.46	2	UD	−
12	66	III	+	0.04	6	−	23.97
13	47	III	−	0.12	4	−	7.65
14	53	III	+	0.22	5	−	13.97
15	56	III	+	0.12	4	−	1.02
16	53	III	+	0.14	4	−	5.75
17	63	III	−	0.12	3	−	5.05
18	51	III	+	0.26	4	−	9.14
19	59	III	+	1.00	6	+	−
20	44	III	−	1.39	5	+	3.42
21	64	II	−	1.86	5	−	−
22	53	I	−	20.96	4	−	2.05
23	53	I	−	0.30	5	+	37.47
24	57	I	−	4.23	6	UD	−
25	45	I	−	0.90	3	−	22.19
26	42	II	−	2.98	4	−	4.32
27	44	II	−	7.04	2	−	12.42
28	67	III	−	8.69	2	−	1.30
29	52	III	−	1.22	0	+	18.72
30	48	III	+	1.71	2	+	3.33
31	54	II	−	9.59	5	−	44.26
32	68	III	−	2.27	4	+	−
33	58	III	+	1.42	4	+	7.86

Relative *miR-34a* expression was normalized with the median.Copy number change could not be determined in two patients.FIGO, International Federation of Gynecology and Obstetrics; IHC, immunohistochemistry; UD, undetermined; PMR, percentage of fully methylated reference.

Supplementary Table 2: Primer list

IL-6R	Forward Primer	5′-GTG CGT CGC CAG TAG TGT-3′
	Reverse Primer	5′-TCA GGC TGC AAG ATT CCA CAA C-3′
GAPDH	Forward Primer	5′-GAC AGT CAG CCG CAT CTT CTT T-3′
	Reverse Primer	5′-ACC AGA GTT AAA AGC AGC CCT-3′
Promoter of *miR-34a*	Forward Primer	5′-CCG CGC GAT CCG AAT-3′
	Reverse Primer	5′-GGA TGA GGA TTA GGA TTT CGT GTT-3′
COL2A1	Forward Primer	5′-TCT AAC AAT TAT AAA CTC CAA CCA CCA A-3′
	Reverse Primer	5′-GGG AAG ATG GGA TAG AAG GGA ATA T-3′
